# Cortical cerebral blood flow in ageing: effects of haematocrit, sex, ethnicity and diabetes

**DOI:** 10.1007/s00330-019-06096-w

**Published:** 2019-03-18

**Authors:** Lorna A. Smith, Andrew Melbourne, David Owen, M. Jorge Cardoso, Carole H. Sudre, Therese Tillin, Magdalena Sokolska, David Atkinson, Nish Chaturvedi, Sebastien Ourselin, Alun D. Hughes, Frederik Barkhof, H. R. Jäger

**Affiliations:** 1 MRC Unit for Lifelong Health and Ageing, Department of Population Science & Experimental Medicine, University College London, WC1E 6HX, London, UK; 2 Centre for Medical Imaging, Division of Medicine, University College London, 2nd Floor, Charles Bell House, 43-45 Foley Street, London, W1W 7TS UK; 3 School of Biomedical Engineering and Imaging Sciences, King’s College London, London, SE1 7EH UK; 4 Department of Medical Physics and Biomedical Engineering, University College London, London, NW1 2BU UK; 5 Dementia Research Centre, UCL Institute of Neurology, London, Wc1N 3BG UK; 6 Institute of Healthcare Engineering, University College London, London, UK; 7 Department of Radiology & Nuclear Medicine, VU University Medical Centre, Amsterdam, Netherlands; 8 Department of Brain Repair and Rehabilitation, UCL Institute of Neurology, London, WC1N 3BG UK; 9 Lysholm Department of Neuroradiology, The National Hospital for Neurology and Neurosurgery, University College London, London, WCN1 3BG UK

**Keywords:** Cerebrovascular circulation, Haematocrit, Ageing, Ethnic groups

## Abstract

**Objectives:**

Cerebral blood flow (CBF) estimates from arterial spin labelling (ASL) show unexplained variability in older populations. We studied the impact of variation of haematocrit (Hct) on CBF estimates in a tri-ethnic elderly population.

**Materials and methods:**

Approval for the study was obtained from the Fulham Research Ethics Committee and participants gave written informed consent. Pseudo-continuous arterial spin labelling was performed on 493 subjects (age 55–90) from a tri-ethnic community-based cohort recruited in London. CBF was estimated using a simplified Buxton equation, with and without correction for Hct measured from blood samples. Differences in perfusion were compared, stratified by sex, ethnicity and diabetes. Results of Student’s *t* tests were reported with effect size.

**Results:**

Hct adjustment decreased CBF estimates in all categories except white European men. The decrease for women was 2.7 (3.0, 2.4) mL/100 g/min) (mean (95% confidence interval (CI)), *p* < 0.001 *d* = 0.38. The effect size differed by ethnicity with estimated mean perfusion in South Asian and African Caribbean women found to be lower by 3.0 (3.6, 2.5) mL/100 g/min, *p* < 0.001 *d* = 0.56 and 3.1 (3.6, 2.5) mL/100 g/min), *p* < 0.001 *d* = 0.48, respectively. Estimates of perfusion in subjects with diabetes decreased by 1.8 (2.3, 1.4) mL/100 g/min, *p* < 0.001 *d* = 0.23) following Hct correction. Correction for individual Hct altered sample frequency distributions of CBF values, especially in women of non-European ethnicity.

**Conclusion:**

ASL-derived CBF values in women, non-European ethnicities and individuals with diabetes are overestimated if calculations are not appropriately adjusted for individual Hct.

**Key Points:**

• *CBF quantification from ASL using a fixed Hct of 43.5%, as recommended in the ISMRM white paper, may lead to erroneous CBF estimations particularly in non-European and female subjects*.

• *Individually measured Hct values improve the accuracy of CBF estimation and, if these are not available, an adjusted value according to gender, ethnicity or diabetes status should be considered*.

• *Hct-corrected ASL could be potentially important for CBF threshold decision making in the fields of neurodegenerative disease and neuro-oncology*.

**Electronic supplementary material:**

The online version of this article (10.1007/s00330-019-06096-w) contains supplementary material, which is available to authorized users.

## Introduction

Arterial spin labelling (ASL) is a magnetic resonance imaging (MRI) technique increasingly used in research and clinical settings to calculate cerebral blood flow (CBF) non-invasively [1]. It has been recognised that ASL can provide an early biomarker for dementia, cognitive decline and small vessel disease [2–8]. Despite statistical differences between groups, e.g. Alzheimer’s disease, mild cognitive impairment and normal ageing, clinical applications have been hampered by unexplained inter-subject variability [9, 10].

Deriving quantitative perfusion values from the raw MRI signal requires the application of a model containing several assumptions that relate to physiological properties of the blood and tissues. The white paper recommendations of the International Society for Magnetic Resonance in Medicine (ISMRM) and the European Consortium for ASL in Dementia propose pseudo-continuous ASL (PCASL) with a single post-labelling delay (PLD) and slice-timing correction, and the application of a simplified Buxton equation for quantification of CBF [11, 12]. In this model, 1650 ms is recommended as the longitudinal relaxation time of blood (T1_blood_) at 3 T. This has been derived from a linear relationship between haematocrit (Hct) and blood T1 found in experiments under appropriate physiological conditions [13], assuming an average adult Hct of 43.5% [11]. However, there are well-recognised sources of variation in Hct between and within populations that may render CBF estimation inaccurate if they are excluded from the model [9, 10].

Hct varies by sex with females typically having lower Hct than males [14]. Most studies show a lower Hct in blacks than Asians compared with whites [15, 16], although an earlier study found this varied according to age and was not applicable in some younger subjects (men aged 15–24) [17]. Higher Hct levels have been associated with obesity [14] and have also been associated with risk of developing diabetes [18]. Diabetic patients with long-standing disease may have decreased Hct, possibly due to diabetic nephropathy causing erythropoietin deficiency [19], or malabsorption of vitamin B_12_ as a side-effect of long-term treatment with metformin [20].

The purpose of this study was to identify the variability in cortical CBF using ASL, to investigate the influence of Hct on the estimation of CBF and to determine how this impacts on the associations of CBF with age, sex, ethnicity and diabetes.

## Materials and methods

### Study population

Subjects (*n* = 493, 40% female, age range 55–90) were recruited from the Southall and Brent Revisited (SABRE) study. Approval for investigations was obtained from the Fulham Research Ethics Committee (ref: 14/LO/0108) and participants gave written informed consent. SABRE is a longitudinal study principally investigating cardio-metabolic disease in a tri-ethnic population cohort; details have been published elsewhere [21]. Briefly, participants were community-dwelling elderly men and women and resident in the north and north-west London at the commencement of the SABRE study in 1988. Ethnicity was defined based on the country of origin of all four grandparents. Spouses or significant others were invited to participate in a clinical visit, commencing in 2014. An additional booster sample of African Caribbeans from the same area of London was recruited to increase numbers and therefore enhance the power of the study. Data for this study were acquired between August 2014 and October 2016.

### Investigations

Participants underwent all examinations on the same day as MRI. Participants were requested to consume no more than an early light breakfast prior to arrival at the clinic. Weight and height were measured using a Tanita Pro BC-418 Body Composition Analyser and a Seca 216 Stadiometer, respectively, and body mass index (BMI) was calculated. Supine central blood pressure (BP) and heart rate (HR) were measured using a Pulsecor device after 5-min rest, in both arms. BP was calculated as the average of the final two of three measurements in the left arm unless the difference between arms was > 10 mmHg in which case the arm with the higher BP was used. Mean arterial pressure (MAP) was calculated using a Pulsecor device (www.uscom.com.au) to measure the arterial pressure waveform during suprasystolic brachial cuff inflations. Hct was measured using an impedance-based, direct current sheath flow method (Sysmex XE-2100) from a venous blood sample drawn on the same morning as the MRI examination. Blood was also analysed for glycosylated haemoglobin (HbA1c), serum total cholesterol, high-density lipoprotein (HDL) cholesterol and triglycerides. Low-density lipoprotein (LDL) cholesterol was calculated using the Friedewald equation.

### Diabetes mellitus

Diabetes status was determined by the following criteria: primary care records of diagnosis or prescription of diabetes medication, patient recall of diagnosis or taking antidiabetic medication, fasting or oral glucose tolerance test or plasma glucose testing from previous SABRE study visits using the WHO recommendations [22] and those with an HbA1c > 47 mmol/mol when tested in the research clinic.

### MRI imaging protocol

All subjects were examined at a single centre, University College Hospital, on a 3 T MRI (Achieva, Philips Healthcare) using an 8-channel phased-array head coil. The protocol included a sagittal T1-weighted 3D-TFE (TR/TE/TI 7/ 3.2/836 ms, flip-angle 18°, voxel size 1 mm^3^) and a transversal 2D pseudo-continuous arterial spin labelling (PCASL) (EPI, TR/TE 4615/15 ms, flip-angle 90°, voxel size 3.75 mm × 3.75 mm × 5 mm, 1-mm slice gap, 20 slices), labelling duration 1800 ms, post-labelling delay 2000 ms, 35 acquisitions, 2 background suppression pulses (1950 ms and 3296 ms after saturation). There were three repetitions of a proton density–weighted image with TR = 9000 ms and no background suppression, but otherwise identical parameters as PCASL were also acquired. Planning was aligned to the anterior commissure-posterior commissure line in the transversal plane orthogonal to the T1w sagittal plane, ensuring coverage of the entire cerebrum including the vertex. The labelling plane was positioned on a phase-contrast survey to identify the vessels.

### MR segmentation and cerebral blood flow processing

Tissue segmentation and region labels were obtained using the Geodesic Information Flows framework [23] (https://github.com/KCL-BMEIS/NiftySeg). This method produces a state-of-the-art segmentation and regional labelling by voxel-wise voting between several propagated atlases, guided by the local image similarity. Grey matter and white matter are defined within the propagated atlases. Segmentations of the grey matter, white matter and cerebrospinal fluid space are resampled to the space of the ASL acquisition making use of the known point-spread function to account for down-sampling induced loss of information.

The determination of CBF maps from ASL data followed the simple derived form for PCASL (Eq. 1) from [11] presented in units of mL/100 g/min using an open-source in-house software package [24] (https://cmiclab.cs.ucl.ac.uk/CMIC/NiftyFit-Release). Thirty-five control, (SC) and label (SL) pairs and 5 proton density (SPD) images were averaged to generate single voxel values for the control and label in Eq. 1, (where λ is the blood/brain partition coefficient (0.9 mL/g), PLD is the post-labelling delay between the end of bolus and the start of imaging (2000 ms), T1_blood_ is the blood T1 relaxation time, α is the labelling efficiency (85%) and τ is the labelling duration (1800 ms). T1_blood_ was calculated based on the formula: T1 = (0.52 × Hct + 0.38)^−1^[13] either with fixed value of 43.5% (corresponding to the standard value of T1 = 1650 ms), which was used in model 1 (CBF_fixed_), or calculated based on the Hct values measured from each participant and used in model 2 (CBF_Hct_). The perfusion difference between CBF models using a fixed Hct and individualised Hct was calculated as an absolute number and as a percentage of the fixed Hct model.

1 $$ \mathrm{CBF}=6000\uplambda\ {\mathrm{e}}^{\mathrm{PLD}/\mathrm{T}1\mathrm{blood}}\ \left(\mathrm{SC}\hbox{--} \mathrm{SL}\right)/\mathrm{SPD}\ \left(2\upalpha\ {\mathrm{T}1}_{\mathrm{blood}}\left(1-{\mathrm{e}}^{-\uptau /\mathrm{T}1\mathrm{blood}}\right)\right)\left[\mathrm{mL}/100\ \mathrm{g}/\min \right] $$

Partial volume correction (PVC) was applied based upon the method used in [25]. Results for segmented cortical grey matter PVC CBF are presented. Cortical grey matter CBF without PVC are presented in supplemental material (Supp Table 1).

### Statistical analysis

Analysis was performed using STATA 14.2 (College Station, TX). All analyses were stratified by sex and ethnicity. Continuous data are presented as mean and standard deviation (SD); categorical data are counts and percentages.

Paired *t* tests were used to test for statistical significance between the two methods of CBF calculation. Pearson’s correlation coefficient was computed to quantify the correlations between Hct and CBF estimates. Comparisons by sex and ethnicity were performed using two-way analysis of variance (ANOVA), followed by individual group-wise comparisons using Fisher’s least significant difference test if ANOVA was significant (*p* < 0.05). Multiple linear regression analyses were performed to assess participant characteristic associations with Hct levels with further adjustment for potential confounders, age, mean arterial pressure, diabetes, HbA1C, LDL cholesterol, HDL cholesterol and BMI, chosen a priori. Sample frequency distributions of CBF calculated with and without correction for individual Hct were analysed using univariate kernel density estimates.

## Results

Five hundred forty-one participants attended MRI. Four hundred ninety-three completed the MRI scan and were successfully processed for CBF*.* Exclusions were due to susceptibility artefacts such as dental work affecting the radiofrequency label (*n* = 12), claustrophobia/inability to tolerate the scan (*n* = 9), segmentation processing errors due to excessive head movement (*n* = 25), pathology (*n* = 1) and scanner artefact (*n* = 1).

### Participant characteristics

Table 1 shows the basic characteristics of the sample stratified by sex and ethnicity. The sample comprised 493 individuals of whom 40% (*n* = 196) were women. Women were younger than men (women mean age ± SD, 69.7 ± 6.4 years; men 72.9 ± 5.2 years; *p* < .001). White Europeans represented 46% of the sample, South Asians 35% and African Caribbeans 19%. A total of 27% of the participants had type 2 diabetes.

**Table 1:** Participant characteristics

		Sex			Ethnicity		
	*N*	All	Men	Women	White European	South Asian	African Caribbean
Sex, *n* (female)	493	493 (196)	297	196	226 (78)	175 (67)	92 (51)**
Age, years	493	71.6 ± 5.9	72.9 ± 5.2	69.7 ± 6.4**	72.1 ± 5.8	70.8 ± 5.6**	72.0 ± 6.7
Diabetes (yes), *n* (%)	493	133 (27)	80 (27)	53 (27)	39 (17)	65(37)	29 (32)**
HbA1c (mmol/mol)	486	41.7 ± 9.2	41.2 ± 8.3	42.5 ± 10.3	39.2 ± 7.9	43.9 ± 8.8**	43.6 ± 11.0**
BMI (kg/m^2^)	489	27.5 ± 4.2	27.1 ± 3.8	28.2 ± 4.8**	27.5 ± 4.1	26.4 ± 3.7**	29.6 ± 4.7**
Haematocrit (% )	493	41.6 ± 3.7	43.0 ± 3.5	39.5 ± 3.0**	42.7 ± 3.6	40.8 ± 3.7**	40.7 ± 3.4**
Total cholesterol (mmol/L)	492	4.7 ± 1.1	4.5 ± 1.0	5.1 ± 1.1**	4.8 ± 1.1	4.5 ± 1.0**	4.8 ± 1.1
LDL (mmol/L)	492	2.5 ± 0.9	2.4 ± 0.9	2.7 ± 0.9**	2.6 ± 0.9	2.3 ± 0.9**	2.5 ± 1.0
HDL (mmol/L)	492	1.60 ± 0.5	1.5 ± 0.4	1.8 ± 0.5*	1.6 ± 0.5	1.5 ± 0.4**	1.8 ± 0.6**
Diastolic BP (mmHg)	480	79.6 ± 8.3	79.6 ± 8.4	79.4 ± 8.0	79.7 ± 8.0	78.6 ± 7.8	80.9 ± 9.5
Heart rate (min^−1^)	481	64.1 ± 11.1	62.4 ± 10.9	66.7 ± 10.9**	64.5 ± 10.8	63.4 ± 11.0	64.7 ± 12.1
Mean arterial pressure (mmHg)	480	97.0 ± 10.0	97.0 ± 10.0	96.9 ± 10.0	96.7 ± 10.1	96.4 ± 9.9	98.6 ± 10.0
Anti-hypertensive medications, (yes) *n* (%)	493	283 (57)	183 (62)	100 (51)**	101 (45)	119 (68)**	63 (69)**
Lipid lowering medications, *n* (%)	493	238 (48)	138 (47)	100 (51)	120 (53)	64 (37)**	54 (59)

Data are mean ± standard deviation or number of observations (*n*) (%). *p < 0.05, **p < 0.01 compared to reference (male/white Europeans) by Fisher’s LSD test after two-way analysis of variance (ANOVA)

### Haematocrit

**Fig. 1:**
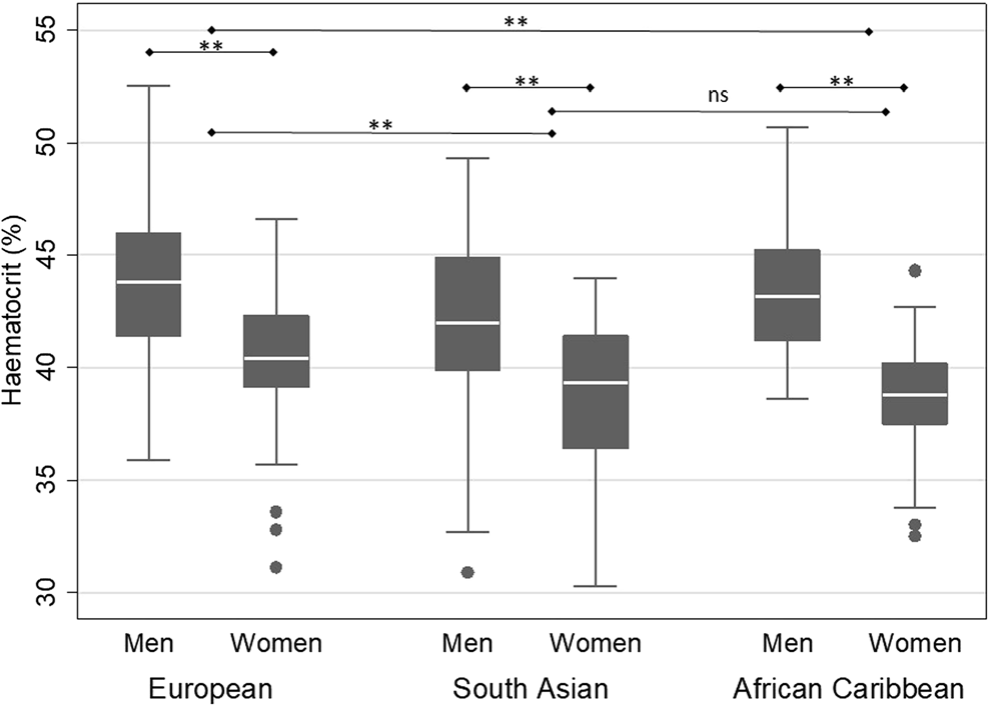
Boxplot showing median, interquartile range, upper and lower adjacent values and outside values for haematocrit (%) by sex and ethnicity. ** = *p* < 0.01 by two-way analysis of variance followed by Fisher’s least significant difference test

The mean (SD) Hct of our sample was 41.6% (3.7). This is lower than the population mean Hct of 43.5% suggested in [11], from which a T1_blood_ of 1650 ms at 3 T had been calculated. South Asian men had a mean Hct of 42.0% (3.6), lower than either white European (43.8% (3.4)) or African Caribbean men (43.0% (3.5)). Both South Asian (38.8% (3.1)) and African Caribbean women (38.8% (2.4)) had lower mean Hct than white European women (40.6% (3.0)) (Fig. 1). Age was not associated with Hct (*r* = 0.06, *p* = 0.2).

### Cerebral blood flow

**Fig. 2:**
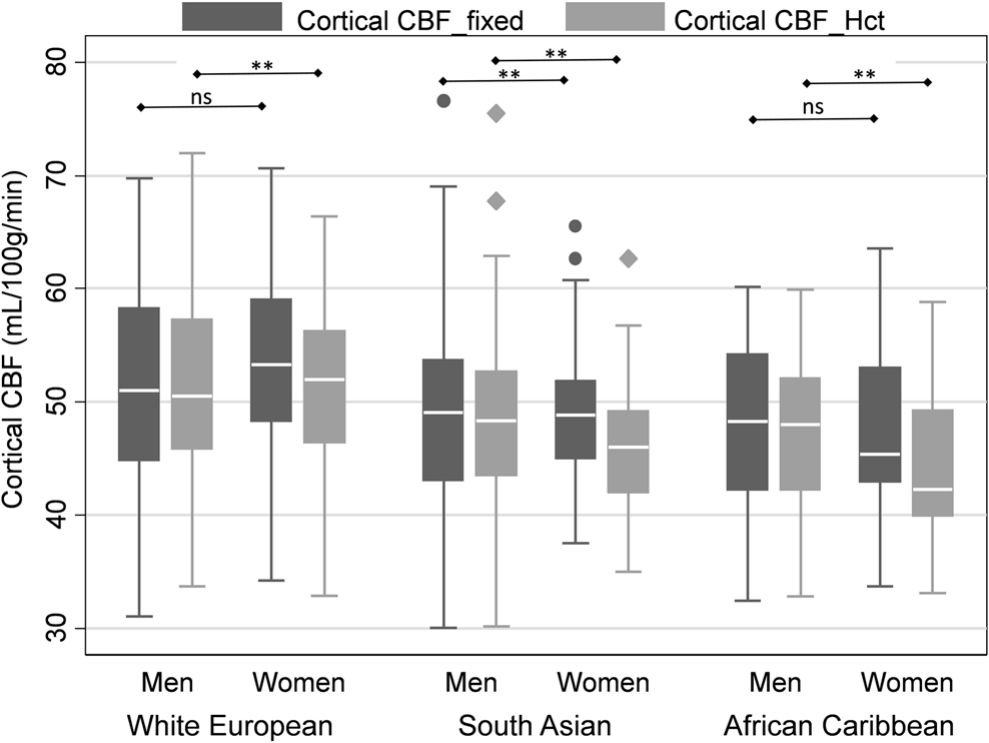
Boxplot showing median, interquartile range, upper and lower adjacent values and outside values for cerebral blood flow without correction for individual haematocrit (CBF_fixed_) and cerebral blood flow with correction for individual haematocrit (CBF_Hct_) by sex and ethnicity. * = *p* < 0.05, ** = *p* < 0.01 by two-way analysis of variance followed by Fisher’s least significant difference test

Table 2 shows the results for CBF_fixed_ and CBF_Hct_ models stratified by sex, ethnicity and diabetes. As a result of correction for individually measured Hct when calculating T1_blood_, CBF_Hct_ values were lower than CBF_fixed_ values in all categories of sex and ethnicity except for white European and African Caribbean men (Fig. 2).

**Table 2:** Comparison of cerebral blood flow without correction for individual haematocrit (CBF_fixed) and cerebral blood flow with correction for individual hemtocrit (CBF_Hct) by sex, ethnicity and diabetes diagnosis

			CBF_fixed (mL/100 g/min)		CBF_Hct (mL/100 g/min)		Difference (mL/100 g/min)	Mean difference (%)	*p* value	Effect size
		*N*	Mean	SD	Mean	SD	Mean (95% CI)	Mean		*d*
	All	493	50.1	± 7.9	48.8	± 7.3	− 1.3 (− 1.5, −1.1)	− 2.6	< 0.001	0.17
	Men	297	50.0	± 8.3	49.6	± 7.6	− 0.4 (− 0.7, −0.1)	− 0.8	0.005	0.05
	Women	196	50.2	± 7.2	47.5	± 6.8	− 2.7 (− 3.0, − 2.4)	− 5.4	< 0.001	0.38
White European	All	226	51.8	± 8.3	51.1	± 7.5	− 0.7 (− 1.0, − 0.4)	− 1.4	< 0.001	0.09
	Men	148	51.1	± 8.6	51.1	± 7.7	0.0 (− 0.5, 0.4)	0.0	0.9	0.00
	Women	78	53.2	± 7.6	51.1	± 7.0	− 2.1 (− 2.6, − 1.6)	− 4.0	< 0.001	0.29
South Asian	All	175	49.1	± 7.3	47.3	± 6.7	− 1.8 (− 2.2, − 1.4)	− 3.7	< 0.001	0.26
	Men	108	49.4	± 8.1	48.4	± 7.3	− 1.0 (− 1.5, – 0.6)	− 2.0	< 0.001	0.14
	Women	67	48.7	± 5.8	45.7	± 5.0	− 3.0 (− 3.6, − 2.5)	− 6.2	< 0.001	0.56
African Caribbean	All	92	47.6	± 6.7	45.8	± 6.3	− 1.8 (− 2.3, − 1.3)	− 3.8	< 0.001	0.29
	Men	41	47.7	± 7.0	47.4	± 6.4	− 0.3 (− 0.9, 0.3)	− 0.6	0.3	0.04
	Women	51	47.6	± 6.6	44.5	± 6.0	− 3.1 (− 3.6,-2.5)	− 6.5	< 0.001	0.48
Without diabetes		377	50.5	± 7.8	49.4	± 7.2	− 1.1 (− 1.4,-0.8)	− 2.2	< 0.001	0.15
With diabetes		116	49.0	± 8.3	47.2	± 7.5	− 1.8 (− 2.3, − 1.4)	− 3.7	< 0.001	0.23

Data are mean ± standard deviation, except difference (95% confidence interval CI), mean difference (%). *p* values were calculated using a Student’s *t* test. Effect size is Cohen’s *d*

**Fig. 3:**
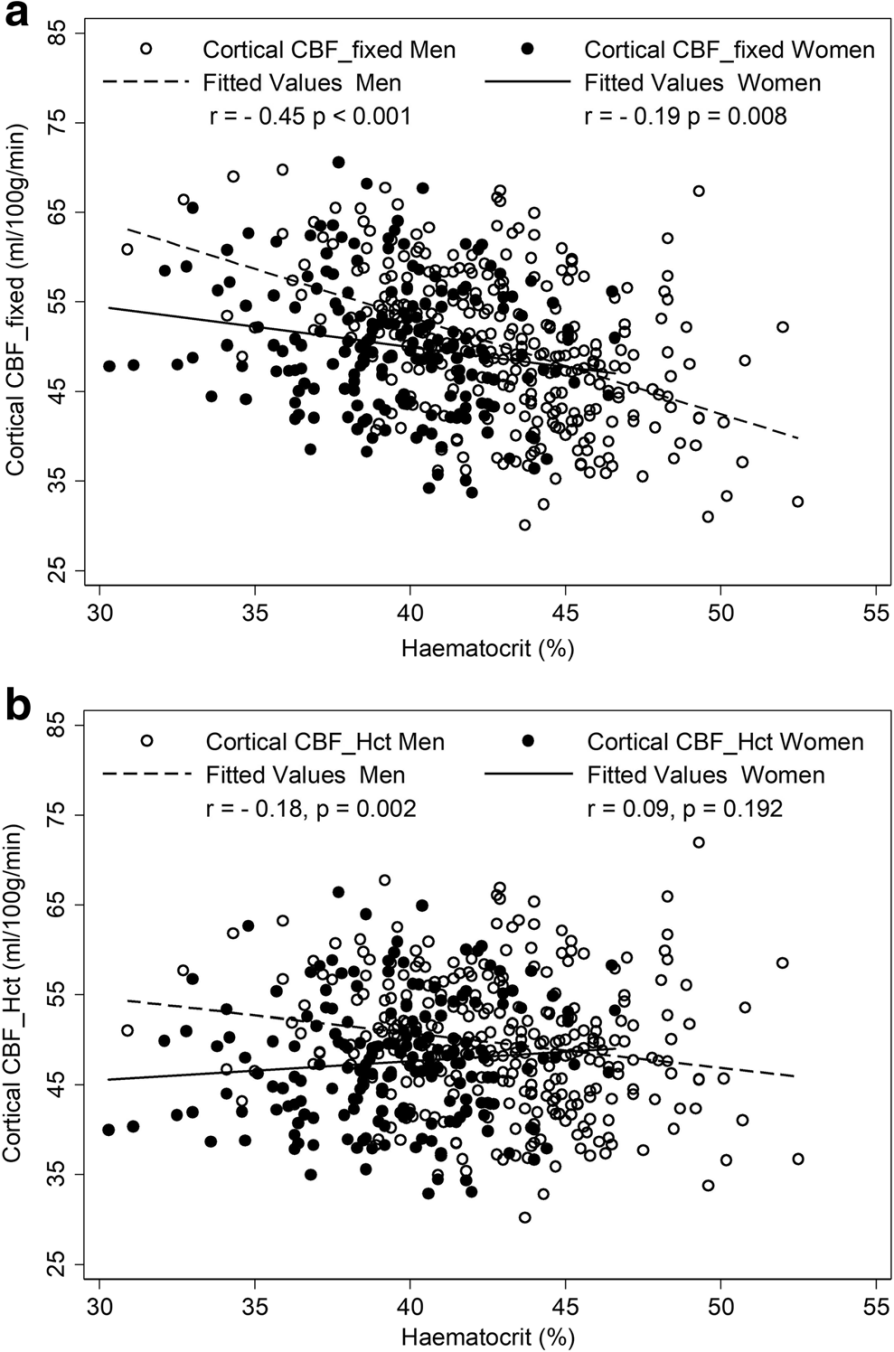
Scatterplots showing the effect of correction for individual haematocrit on the correlation between haematocrit and cortical cerebral blood flow in men and women. Fixed Hct (CBF__fixed_) (**a**); individualised Hct (CBF__Hct_) (**b**)

Figure 3 demonstrates the inverse linear relationship of Hct with CBF_fixed_ and CBF_Hct_ models. This relationship is attenuated with the use of the CBF_Hct_ model although some association of Hct with CBF in men remained (*r* = − 0.18, *p* = 0.002). Further adjustment for potential confounders of mean arterial blood pressure, BMI, diabetes and dyslipidemia did not affect this relationship when entered in a regression model (β = − 0.3, CI − 0.6, − 0.05 mL/100 g/min, *p* = 0.020) (Supp Table 2). (Note: model 2 has fewer subjects due to missing cardiovascular data.)

**Fig. 4:**
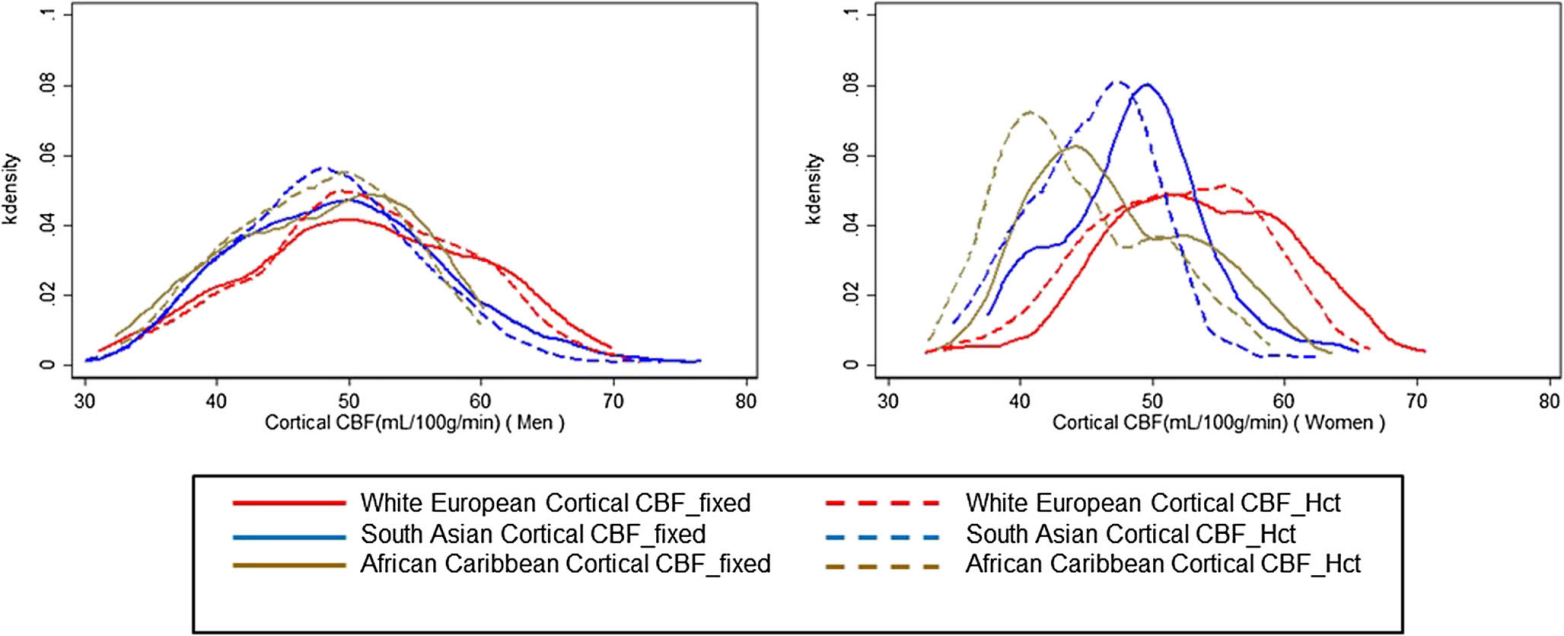
Kernel density (kdensity) plots of CBF without correction for individual haematocrit (CBF_fixed_) and CBF with correction for individual haematocrit (CBF_Hct_) by sex and ethnicity

**Fig. 5:**
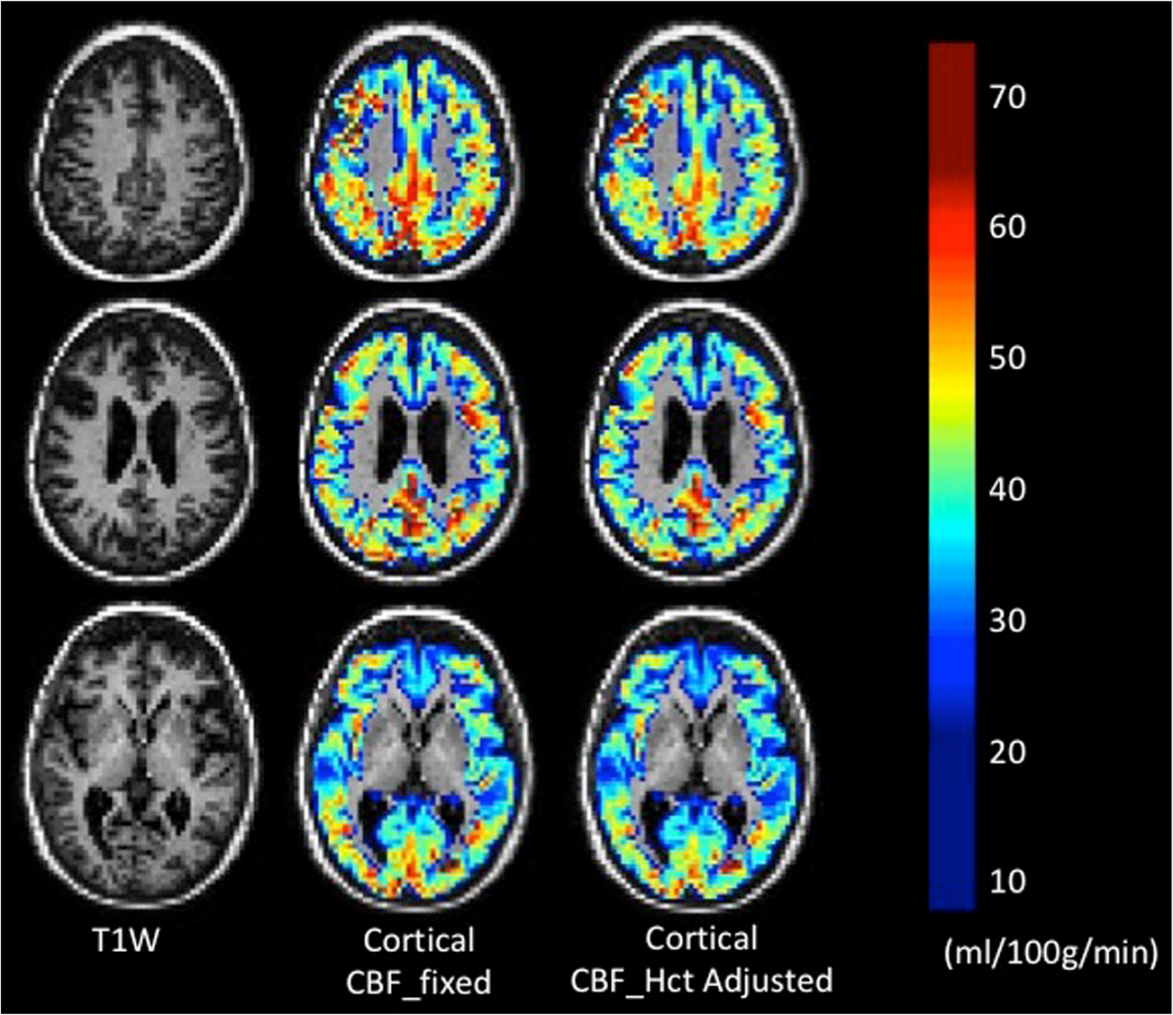
CBF maps overlaid on segmented T1w without and with adjustment for measured haematocrit. The subject was a white European woman with an haematocrit of 37.3%

Introduction of individualised Hct into CBF estimation altered the distributions of cortical CBF values in this elderly population for all ethnicities (Fig. 4). The mean perfusion difference between models was greater for women in all ethnic categories than for men, with a mean decrease for women of 2.7 mL/100 g/min (5.4% of the CBF_fixed_ value) (*t* = 17.1; CI 3.0, 2.4 mL/100 g/min; *p* < 0.001, *d* = 0.38). The perfusion difference for African Caribbean women was − 3.1 mL/100 g/min (6.5% decrease from the CBF_fixed_ value) (*t* = 10.4; CI − 3.6, − 2.5 mL/100 g/min; *p* < 0.001, *d* = 0.48), and for South Asian women, it was − 3.0 mL/100 g/min (6.2% decrease from the CBF_fixed_ value) (*t* = 10.9; CI − 3.6, − 2.5 mL/100 g/min; *p* < 0.001, *d* = 0.56). South Asian men were the only male category displaying a significant difference between models with a perfusion difference of − 1.0 mL/100 g/min (2.0% decrease from the CBF_fixed_ value) (*t* = 4.3; CI − 1.5, − 2.0 mL/100 g/min; *p* < 0.001, *d* = 0.14). Figure 5 provides an example of CBF maps illustrating the decrease in CBF estimation of a female subject with an Hct of 37.3%.

The application of PVC removed most of the negative association of CBF with age in both models (CBF_fixed_, *r* = − 0.02, *p* = 0.7; CBF_Hct_, *r* = 0.00, *p* = 0.2). Adjustment for Hct did not significantly alter this relationship.

### Diabetes

**Fig. 6:**
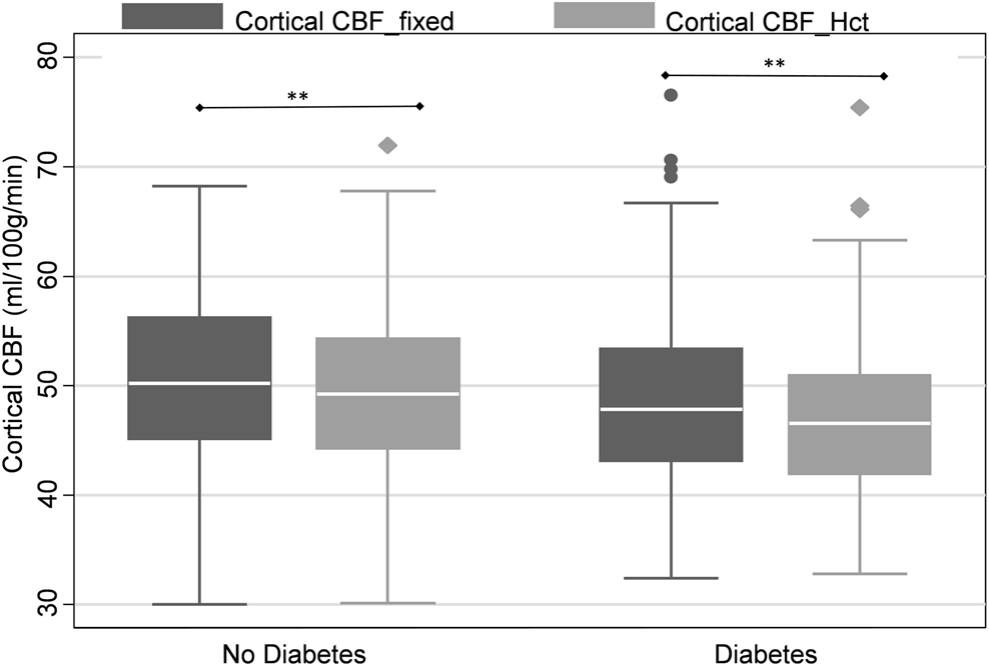
Boxplot showing median, interquartile range, upper and lower adjacent values and outside values for CBF without correction for individual haematocrit (CBF_fixed) and CBF with correction for individual haematocrit (CBF_Hct_) by diabetes status. * = *p* < 0.05, ** = *p* < 0.01 by Student’s *t* test

People with diabetes had a mean (SD) Hct of 40.7% (4.1) compared with 42.0% (3.5) for those without diabetes. The perfusion difference between CBF models of people with diabetes was − 1.8 mL/100 g/min (3.7% of the CBF_fixed_ value) (*t* = 7.8; CI − 2.3, − 1.4 mL/100 g/min; *p* < 0.001, *d* = 0.23), whereas those without diabetes only displayed a difference of − 1.1 mL/100 g/min (2.2% of the CBF_fixed_ value) (*t* = 8.4; CI − 1.4, − 0.8 mL/100 g/min, *p* < 0.001, *d* = 0.15) (Fig. 6).

## Discussion

This study has shown that Hct levels differ according to sex and ethnicity and that this influences CBF estimated from ASL. Importantly, failure to adjust T1_blood_ according to sex and ethnic variation in Hct leads to a significant overestimation of CBF in women and non-European populations. Although other studies have investigated the effect of Hct on CBF estimation with ASL in sickle cell [26] and neonatal [27] groups; this is to the best of our knowledge, the first study to address this in a community-based elderly multi-ethnic population.

A study by Parkes et al [28] using continuous ASL found females had higher grey matter CBF than males by 13%. It seems likely that failure to measure individual Hct influenced these observations. We found a similar difference in CBF between white European men and women (+ 9.1% for women) when neither Hct nor PVC was accounted for, but this difference was completely abolished by correction for Hct and PVC. Following correction for PVC and Hct, CBF was slightly lower for women than for men in South Asian (− 5.6%) and African Caribbean (− 6.0%) ethnicities. This finding is broadly aligned with an ^15^O PET study where gender differences in CBF evident in younger subjects were not significant in subjects older than 65 years [29]. It has been suggested that gender differences in young subjects are due to high oestrogen levels in females causing CBF fluctuations during the menstrual cycle [30]. Our large elderly sample scanned with age-appropriate parameters such as a long PLD and with CBF calculated using individualised values is likely to provide a more accurate reflection of true physiological differences than studies that have used a ‘one size fits all’ protocol.

We also demonstrated that the utilisation of individually measured Hct in the calculation of T1_blood_ reduced the inverse correlation between CBF and Hct. However, some association remained in men even after adjustment for mean arterial blood pressure, diabetes and dyslipidemia. Increased CBF is an expected response to decreases in haemoglobin (and thus Hct) as a mechanism to sustain the cerebral metabolic rate of oxygen levels (CMRO_2_) as demonstrated by Ibaraki et al [31]. It has also been suggested that higher Hct levels and concomitant increases in blood viscosity may influence capillary flow due to alterations in functional shunting [32]. The higher Hct levels found in men may provide an explanation why we found some association between Hct and CBF only in men as increasing Hct increases blood viscosity in a non-linear relationship, and it has been suggested that increased viscosity decreases CBF [33].

Lower values of Hct in those with diabetes indicate that further investigation using larger samples is warranted to investigate the interactions of sex, ethnicity, Hct and the effect of diabetes on CBF.

Investigation of the association of age and CBF, which had yielded conflicting evidence in previous studies [10, 28, 34], was not the main aim of our study. However, it is noteworthy that we found an association between age and CBF in the non-partial volume-corrected data which disappeared following partial volume correction. A likely explanation for this is the decreased brain volume in older subjects which makes the ASL perfusion data more prone to partial volume effects with non-perfused CSF, thereby leading to an artificial CBF decrease. However, PVC may introduce overestimation of cortical CBF particularly in examinations hampered by head movement or poor SNR, either in the T1 structural images used for segmentation or in the ASL acquisition which as a subtraction technique is particularly susceptible to movement artefact. Some movement inevitably remains in a large population study of elderly individuals and this may be a potential source of under- or overestimation of partial volume-corrected CBF in our cohort.

Despite a more diverse ethnic sample, our cortical grey matter CBF values are comparable with previous studies using PCASL on elderly, community-dwelling populations [6, 7]. PCASL has some limitations when applied to elderly subjects. One factor affecting CBF quantification is blood velocity relative to the labelling plane. Any discrepancies from the expected range of blood velocities due to vascular pathologies such as internal carotid stenosis or vessel tortuosity might result in reduced labelling efficiency and therefore CBF underestimation. Our study used 2D PCASL, rather than a 3D technique, which may be liable to decreased SNR due to longer T1 blood relaxation in slices near the vertex due to sequential slice acquisition [35], despite using slice-timing correction. Inefficiency of background suppression pulses during multiple slice acquisitions may also have affected the ASL signal.

The main strength of this study was its large community-based, elderly, ethnically diverse sample studied in a single centre so that all protocols were consistently applied throughout. Limitations of the study include a potential selection bias towards healthy individuals who were willing and able to attend the research clinic. Another limitation was the absence of direct T1_blood_ measurements, although this was mitigated by the use of a previously published model to account for the effect of Hct [13]. Hct is the main determinant of T1_blood_, but other factors such as serum ferritin and HDL cholesterol may also contribute to a minor extent [36]. Measurement of arterial rather than venous Hct would have been more appropriate to estimate CBF. However, measuring arterial Hct in clinic was impractical and it was considered that venous and arterial Hct were closely related and importantly that venous Hct could be sampled consistently [37]. Although T1_blood_ can be easily directly measured in the left ventricle and has lower test-retest variability compared with measured Hct [38], comparable sequences in the brain are more challenging due to partial volume effects, blood velocity and pulsatility in the measured vessel [27, 39].

Our findings suggest that research studies using ASL to measure CBF should routinely measure Hct to adjust T1_blood_, especially in inhomogenous samples. Alternatively, substitution of an appropriate gender- and ethnicity-specific Hct value derived from population group means or direct measurement of T1_blood_ during MRI would improve accuracy of CBF measurement. Further research is warranted into whether adjustment to the Hct value in CBF models to accommodate demographic and pathological differences provides stronger associations with cerebrovascular disease, dementia and cognitive decline than previous models using a fixed mean Hct value. Results from previous studies may need to be interpreted with caution where there are ethnic, gender and pathological differences in the sample. Such an approach may improve early risk assessment in ethnic groups and identify potentially vulnerable groups such as those with known vascular or metabolic disease.

In conclusion, we demonstrated that CBF values obtained from ASL using a fixed Hct mean may lead to systematic errors, resulting most frequently in an overestimation of CBF in female subjects and non-Caucasian ethnicities. It is important to be aware of this when using CBF threshold values to assess disease status or severity. This applies not only to the discrimination between normal ageing and a neurogenerative disease such as AD but could potentially also influence the discrimination between high- and low-grade brain tumours or determination of penumbral threshold values in stroke. We therefore argue that whenever possible, individualised measures of Hct should be included in CBF calculations by ASL.

## Electronic supplementary material

ESM 1 (DOCX 24 kb)

## Abbreviations

ASL: Arterial spin labelling
BMI: Body mass index
CBF: Cerebral blood flow
CMRO_2_: Cerebral metabolic rate of oxygen
HbA1c: Haemoglobin A1c (glycated haemoglobin)
Hct: Haematocrit
HDL cholesterol: High-density lipoprotein cholesterol
LDL cholesterol: Low-density lipoprotein cholesterol
PCASL: Pseudo-continuous arterial spin labelling
PLD: Post-labelling delay
PVC: Partial volume correction
