# Supplementary material for: Cortical cerebral blood flow in ageing: effects of haematocrit, sex, ethnicity and diabetes
Source: Eur Radiol. 2019 Mar 18;29(10):5549–58. doi: 10.1007/s00330-019-06096-w (PMC6719435; doi:10.1007/s00330-019-06096-w)
Supplement: Supplementary file 1 — (DOCX 24 kb) [file 330_2019_6096_MOESM1_ESM.docx]

# Supplemental Material

|  | |  |  | **CBF_fixed**  **(mL/100g/min)** | | **CBF_Hct**  **(mL/100g/min)** | | **Difference (mL/100g/min)** |  | **Mean Difference (%)** | ***P-*value** | **Effect Size** |
| --- | --- | --- | --- | --- | --- | --- | --- | --- | --- | --- | --- | --- |
|  | |  | *n* | *mean* | *SD* | *mean* | *SD* | *mean (95% CI)* | | *mean* |  | *d* |
|  | | All | 493 | 37.8 | ± 7.0 | 36.6 | ± 6.3 | -1.2 (-1.4, -1.0) | | -3.2 | <.001 | 0.17 |
|  | | Men | 297 | 36.9 | ± 7.3 | 36.5 | ± 6.7 | -0.4 (-0.6, -0.1) | | -1.1 | 0.004 | 0.05 |
|  | | Women | 196 | 39.1 | ± 6.3 | 36.7 | ± 5.8 | -2.4 (-2.6, -2.1) | | -6.1 | <.001 | 0.39 |
|  |  | | | | | | | | | | |  |
| White European | | All | 226 | 38.7 | ± 7.5 | 38.0 | ± 6.7 | -0.7 (-1.0, -0.4) | | -1.8 | <.001 | 0.09 |
|  | | Men | 148 | 37.5 | ± 7.7 | 37.5 | ± 7.0 | 0.0 (-0.3, 0.3) | | 0.0 | 0.922 | 0.00 |
|  | | Women | 78 | 40.9 | ± 6.8 | 39.0 | ± 6.0 | -1.9 (-2.4, -1.5) | | -4.7 | <.001 | 0.30 |
|  |  | | | | | | | | | | |  |
| South Asian | | All | 175 | 36.9 | ± 6.3 | 35.3 | ± 5.7 | -1.6 (-1.9, -1.2) | | -4.3 | <.001 | 0.26 |
|  | | Men | 108 | 36.5 | ± 6.8 | 35.5 | ± 6.2 | -0.9 (-1.3, -0.5) | | -2.7 | <.001 | 0.14 |
|  | | Women | 67 | 37.5 | ± 5.3 | 34.9 | ± 4.8 | -2.6 (-3.1, -2.2) | | -6.9 | <.001 | 0.52 |
|  |  | | | | | | | | | | |  |
| African Caribbean | | All | 92 | 37.3 | ± 6.5 | 35.7 | ± 5.8 | -1.6 (-2.1, -1.2) | | -4.3 | <.001 | 0.27 |
|  | | Men | 41 | 35.8 | ± 6.8 | 35.5 | ± 6.3 | -0.3 (-0.8, 0.3) | | -0.8 | 0.315 | 0.04 |
|  | | Women | 51 | 38.5 | ± 6.1 | 35.8 | ± 5.5 | -2.7 (-3.2, -2.3)) | | -7.0 | <.001 | 0.47 |
|  |  | | | | | | | | | | |  |
| Without diabetes | |  | 377 | 38.2 | ± 6.8 | 37.2 | ± 6.2 | -1.0 (-1.2, -0.8) | | -2.6 | <.001 | 0.15 |
| With diabetes | |  | 116 | 36.7 | ± 7.5 | 35.1 | ± 6.6 | -1.7 (-2.1, -1.2) | | -4.4 | <.001 | 0.24 |
|  | |  |  |  |  |  |  |  | |  |  |  |

Data are mean ±standard deviation, except difference (95% confidence interval CI), mean difference (%). *P* values were calculated using a Student’s t-test. Effect size is Cohen’s *d*

**Supp Table 1; Comparison of CBF without correction for individual hematocrit (CBF_fixed_) and CBF with correction for individual hematocrit (CBF_Hct_ ) by sex, ethnicity and diabetes diagnosis*:* not partial volume corrected.***.*

|  | **Model 1** |  | **Model 2** |  |
| --- | --- | --- | --- | --- |
| Observations | 493 |  | 466 |  |
| R-squared | 0.26 |  | 0.28 |  |
| **Covariables** | **β coefficient (95% CI)** | ***P* value** | **β coefficient (95% CI)** | ***P* value** |
| Age (years) | -0.055 (-0.10, -0.01) | 0.017 | -0.042 (-0.09 , 0.00) | 0.076 |
| Sex (female) | -3.604 (-4.19 , -3.02) | <0.001 | -3.693 (-4.34 , -3.05) | <0.001 |
| South Asian | -1.799 (-2.45 , -1.14) | <0.001 | -1.669 (-2.36 , -0.98) | <0.001 |
| African Caribbean | -1.178 (-1.88 , -0.48) | 0.001 | -1.154 (-1.87 , -0.44) | 0.002 |
| Diabetes | - |  | -1.149 (-2.14 , -0.16) | 0.023 |
| LDL Cholesterol (mmol/L) | - |  | 0.44 (0.10 , 0.78) | 0.012 |
| HDL Cholesterol (mmol/L) | - |  | -0.068 (-0.77 , 0.63) | 0.85 |
| Mean Arterial Pressure (central)(mmHg) | - |  | -0.009 (-0.04, 0.02) | 0.54 |
| HbA1c (mmol/L) | - |  | 0.033 (-0.01, 0.08) | 0.16 |
| BMI (m/kg^2^) | - |  | -0.018 (-0.09 , 0.05) | 0.62 |
| Constant | 51.438 (47.88 , 55.00) | <0.001 | 51.113 (45.69 , 56.54) | <0.001 |

**Supp Table 2: Multiple linear regression analyses of associations of demographic and cardiovascular risk factors. Hematocrit is dependent variable, model 1 independent variables are age, sex and ethnicity, model 2 is model 1 + independent variables LDL Cholesterol, HDL Cholesterol, mean arterial pressure, HbA1c and BMI.**
